# Kinetics, kinematics, and performance modeling of two arm swing techniques in the swimming kick-start

**DOI:** 10.1038/s41598-026-48558-4

**Published:** 2026-04-16

**Authors:** Stefan Szczepan, Zofia Wróblewska, Daria Rudnik, Faye Perkins, Peter Bodary, Michael Bottom

**Affiliations:** 1 Department of Swimming, Faculty of Physical Education and Sport, Wroclaw University of Health and Sport Sciences, Swimming Pool, 35 Ignacy Jan Paderewski Ave, Wroclaw, 51-612 Poland; 2 Faculty of Pure and Applied Mathematics, Wroclaw University of Science and Technology, Wroclaw, Poland; 3 Department of Recreation and Tourism, Faculty of Physical Education and Sport, Wroclaw University of Health and Sport Sciences, Wroclaw, Poland; 4 Department of Health & Human Performance, University of Wisconsin- River Falls, River Falls, United States of America; 5 School of Kinesiology, University of Michigan, Ann Arbor, United States of America; 6 Men’s and Women’s Swimming and Diving Unit, University of Michigan, Ann Arbor, United States of America

**Keywords:** Biomechanics, Swimming, Start performance, Arm movement, Engineering, Physics

## Abstract

This experimental study investigated the effect of two arm swing techniques on kinematic and kinetic variables during the swimming kick-start. Twelve elite male swimmers performed starts using forward and butterfly arm swing techniques. Kinematic and kinetic data were collected using a Kistler Performance Analysis System. Data were analyzed for variables calculated from ground reaction force (GRF) and block, above the water, and underwater performance. The results revealed that work, power, and most of the force parameters measured higher values for the butterfly arm swing technique (*p* < 0.05). The butterfly arm swing was also advantageous regarding resultant underwater and vertical take-off velocities (*p* < 0.05). The butterfly arm swing technique demonstrated a significant correlation (*p* < 0.05) between the 15 m swimming time trial and additional parameters, including take-off horizontal velocity, power, force on the block, entry distance, and performance at 5, 7.5, and 10 m of swimming. Regression analysis indicated the combination of different predictors of the time to 15 m, including some GRF metrics, and above the water and underwater parameters for both types of arm swing. The butterfly arm swing produced a significantly shorter start time over the distance from 7.5 m to 15 m than the forward arm swing (*p* < 0.05). However, this is not necessarily related to the early phase of the start because no significant differences between analyzed dives were found at the 5 m time (*p* = 0.056).

## Introduction

A well-executed start can significantly reduce a swimmer’s overall race time, making it an important factor in determining the outcome of a competition^1^. Over the years, the swim start from the platform technique has evolved from the grab start, track start, to kick start or their variations^2^. With changes in starting techniques, their differences and determinants have been studied frequently^3^. The start time from the starting signal to the 15 m mark can be impacted by many factors, such as the swimmer’s mechanical power of the lower limbs and agility, starting conditions, construction of the starting platform, and starting technique^4^. Multiple research studies have found that actions on the block critically influence not only the push-off phase^5^ and flight phase^6^ but also the subsequent water entry angle^7^ and underwater actions^3^. Scientists and coaches have also reported several other determinants of the swim start, including foot placement on the platform^8^, the position of the center of gravity at take-off (front and rear-weighted)^9^, and arm movements during the start^10^.

The importance of upper limb movements for improving vertical jump and horizontal jump performance on dry land has been demonstrated by increases in athletes’ force impulse and take-off velocity^11,12^. The increased take-off velocity resulted from a complex sequence of biomechanical events that allowed the arms to accumulate energy early in the movement and subsequently transfer it to the muscles of the lower limbs during the later phase of take-off^13^. Consequently, various arm swing techniques have been introduced in competitive swimming. The first variation can be characterized as a conventional style ‘straight backswing’ in which the swimmer swings the arms back and then forward after the starting signal^14^. In the second method, characterized as a ‘butterfly arm swing,’ the swimmer swings the arms similar to a butterfly arm recovery^15^. The butterfly arm swing was a variation on the ‘circular backswing’ start that was used before the grab start appeared in competition^15^. Third, a more common technique, is ‘forward arm swing’ characterized by simply releasing the arms forward^16^.

Maglischo^15^ has highlighted the advantages of arm swing techniques like the straight backswing and butterfly arm swing over the traditional starts with no arm swing, noting differences in flight phase duration and angle of water entry. Furthermore, temporal variables describing styles of swim start, i.e., conventional style (straight backswing), grab start, bunch start, and track start, have been compared^17^, but there was insufficient evidence detailing how these techniques affected performance beyond the initial start phase. As time passed, arm swings were modified toward a one-handed track start^18^. Additionally, the kinematics and kinetics of styles of swim start, i.e., flat start using a flatter body position, pike start with large take-off and entry angles, flight start with the arms directly in front of the head, and Volkov start with the arms back during the leg impulse, have been examined^10^.

Previous studies conducted several decades ago on start techniques, particularly focusing on arm swing variations^14,17^, have significant gaps and inconsistencies in their results largely due to the use of outdated methodologies. In turn, available data in academic books relied heavily on only theoretical considerations^15^. Other researchers used comparative methods with a single type of analysis, which was insufficient to address the complexity of swim start performance^18^. In turn, previous video analysis did not fully capture the mechanics of the butterfly arm swing, including force production and impulse generation, which contribute to take-off velocity and start performance^10^. Still others, although they distinguished in their studies between the counter-movement backward arm swing and the no counter-movement backward arm swing techniques, did not investigate which technique produced optimal start performance^19^. To date, there is a lack of comprehensive understanding of the biomechanical differences in the swim start resulting from variations in arm movement.

This is the first study to investigate kinematic and kinetic differences between forward and butterfly arm techniques with a broad statistical approach across three domains, group comparison analysis, correlation, and predictive modeling. Demonstration of differences and understanding the relationships between kinematic and kinetic parameters and start results across different arm swing techniques could help identify areas to achieve incremental gains in start performance. In addition, regression modeling may provide valuable information on key factors that best predict swim start performance, potentially highlighting the practical implications of this study.

The purpose of this study was threefold. The first objective was to investigate differences in kinematic and kinetic variables between the forward and butterfly arm techniques using the kick-start among elite swimmers. The secondary objective was to determine the relationships between selected kinematic and kinetic variables of both arm swing techniques and start performance, expressed as the time to 15 m. The third objective was to evaluate which variables would most influence the overall start performance (time to 15 m) during the two different arm swing techniques.

This study hypothesized that the butterfly arm swing would significantly affect the greater peak force and take-off velocity when compared with the forward arm swing in elite swimmers, and that swimmers would elicit faster times over 15 m when using the butterfly arm technique. Furthermore, for the butterfly arm swing, these key kinematic and kinetic variables will strongly correlate with and serve as meaningful predictors of start performance.

## Methods

### Participants

Twelve elite male swimmers were recruited for this study, with one multi-medalist Olympic champion, and the remaining subjects were BIG10 Conference representatives, having competed at the National Collegiate Athletic Association (NCAA) Division I level. All participants were members of the university swimming program and belonged to the sprint group. They had a World Aquatics score of 771.66 ± 98.03 points at the time of data collection (range from 1 to 1000 points)^20^. The average sports level of the participants was at the second threshold according to the standardized performance classification model in swimming (between 650 and 800 points)^21^. Similarities of the swimmers’ somatic parameters (BMI, body weight, body height) were used as an objective basis to compare their potential in terms of start and swimming performance^22^. It was decided that an objective assessment of the test group homogeneity would be conducted with a Grubbs’ outlier test using the following formula^23^(1):

1 $${\rm g = max[x_i-\bar{x}] \cdot \left(SD\right)^{-1}}$$

where: the maximum of the absolute differences between the values x_i_ and the sample means x̄ is divided by the standard deviation (SD) of the sample.

The statistic g indicates an outlier (if g> critical value (g_crit_)). An extract of the critical value for *n* = 12 (sample size) and at significance α level = 0.05 was g_crit_=2.41. The results of the test found that the group was homogeneous in terms of somatic parameters (BMI, body weight, body height) and sports level (best 50 m free time in short course). The participants’ characteristics and the outcome of Grubbs’ outlier test are detailed in Table 1.

**Table 1:** Participant characteristics.

Variables	M	SD	95% CI	min	max	g
Age (yrs)	21.17	5.51	17.66–24.66	18.00	38.00	3.06*
BMI (kg⸱(m^2^)^−1^)	25.76	4.63	22.81–28.70	21.02	33.79	1.73
Body weight (kg)	82.03	5.94	78.25–85.80	71.91	92.22	1.72
Body height (cm)	180.95	18.89	168.94–192.95	155.44	201.16	1.35
Competitive experience in swimming (yrs)	12.58	6.39	8.52–16.64	5.00	30.00	2.73*
Best 50 m freestyle time (short course) (s)	22.16	0.92	21.57–22.74	20.85	23.27	1.42

Note: M - mean, SD - standard deviation, 95% CI − 95% confidence interval. g - Grubbs’ Outlier Test, *Significant (*p* < 0.05) Grubbs’ outlier test outcome g> g_crit_=2.41.

The inclusion criteria were (a) at least five years of competitive swimming experience at the national level, (b) having the front crawl as their primary competition stroke, (c) being able to attend all testing sessions scheduled in this study (d) no absolute contraindication to exercise testing as recommended by the American Heart Association guidelines^24^. Swimmers were excluded if they had sustained an injury or disease three months before the beginning of the study; there were no exclusions. Swimmers were instructed to maintain their normal lifestyle and diet and refrain from any additional exercise external to the test prescribed in the study. All swimmers were reportedly free of drugs, medications, or dietary supplements known to influence physical performance^25^. Subjects were informed of the benefits and purpose of the study, the procedure that would be used, and any potential risks. All subjects provided written informed consent under institutional regulations. Informed consent was also obtained to publish the images in an online open-access publication. They were informed they could withdraw at any time. The study experienced no withdrawals. This study was approved through the institutional ethics committee (approval code: 06 2022) and was adhered to throughout the investigation with the ethical standards of the Declaration of Helsinki and good clinical practice guidelines.

At the time of the investigation, the swimmers were typically participating in 11 swimming sessions (16 h of aquatic workouts/about a total distance of 60 km) and 11 dryland sessions (approximately a total of five hours) per week under the direction of the same coach. The participants used different types of arm swings during their daily basic and competition routines. Therefore, the participants undertook familiarization sessions one week before testing to become accustomed to the two different arm movement types evaluated in this investigation.

### Procedure

Before testing, the participants completed a standard warm-up on land and in water to ensure the reproducibility of the study, prepare for maximal effort, and reduce the chance of injury. The warm-up on land included arm swings in various positions (20 reps); walkout with twist (5 reps); elastic band pull-apart (15 reps); scapula push-ups (10 reps); scapula pull-ups (10 reps); 3 kg med ball throws (5 reps); and squat jumps (15 reps). The warm-up in water covered a total distance of 600 m and consisted of the following parts: 100 m swim (easy pace); 3 × 100 m swim (kick/drill); 4 × 25 m (12.5 m 90% of the 50 m race pace followed by 12.5 m easy); and 100 m easy swim.

**Fig. 1:**
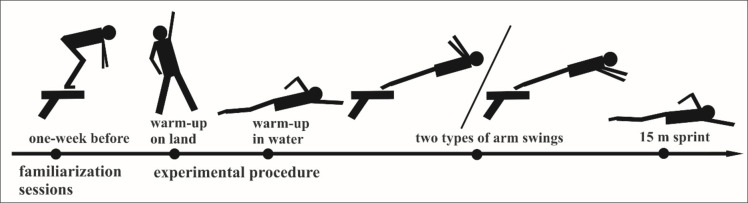
Graphical presentation of the experimental procedure.

This study used a randomized crossover design with a single group of participants, with rest periods between trials. The main experimental procedure consisted of a series of starts with two different arm movement types (forward arm swing - A and butterfly arm swing - B). In total, each swimmer performed four starts (two starts for each arm movement type), and trials were performed in an interspersed sequence (ABAB or BABA) during a one-day testing session. All starts ended with a 15 m front crawl swim with a free-breathing pattern as if in competition. They were verbally encouraged before and during the test to maintain maximal effort. The number of trials was minimized to avoid fatigue and to better predict maximal effort. The rest time between each trial was six minutes to achieve total recovery^26^. According to Bogdanis^27^, a six-minute recovery is sufficient to restore the phosphates source and allow the subject to perform maximal effort in consecutive trials. To minimize any overtraining effects during the experiment, swimmers avoided stressful training during the days before the test. All trials were performed with training swimwear preferred by the swimmers. During data collection, the participants stayed in the first two lanes of the pool, to which others had no access. The schematic diagram with a chronology of testing is presented in Fig. 1.

### Data collection

**Fig. 2:**
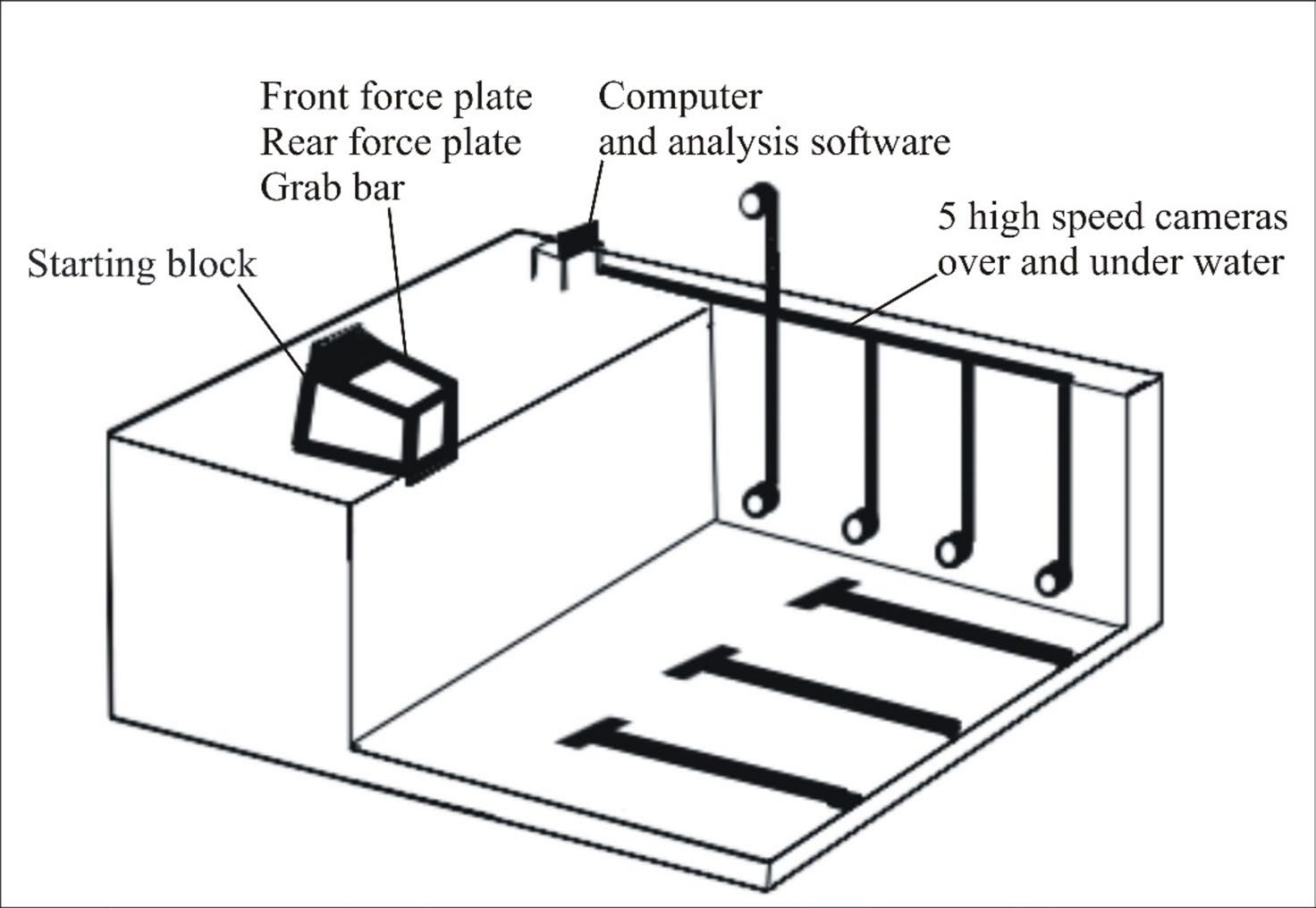
Performance Analysis System (Kistler Instrumente AG, Switzerland) set up in the swimming pool.

Each start was tested using the Performance Analysis System Type 9691A1, including the Starting Block System Type 55,154,262 with the Force Plate Type 9260AA, and a series of five high-speed video cameras (one above the water and four underwater) (Kistler Instrumente AG, Switzerland). The Kistler System is a proprietary system developed by Kistler Instrumente and consists of an instrumented starting block with the same dimensions as the Omega OSB11 starting block that is used at all major swimming events^28^. The multi-component device recorded a dynamic and quasi-static measurement of the three orthogonal components of a force, vertical (Fz), anterior-posterior (Fy), and medial-lateral (Fx) acting from any direction on the top plate. An instrumented grab bar measured the force exerted by hands and arms. All components include piezoelectric sensors. Five high-speed video cameras captured the swimmer’s motion above the water and underwater at 100 fps. The cameras allowed precise measurement of position and split times over a 15 m distance at high resolution. The data acquisition system provided a frame-by-frame synchronization of the force plate and video data. The software displayed video along with a biomechanical analysis of the kinetic and kinematic variables. The validity and reliability of this system have been previously established by the producer (Kistler Instrumente AG, Switzerland). A lab technician with specialized training with the Kistler System took all measurements. The system was calibrated before the experiment. The schematic setting of devices is provided in Fig. 2.

### Independent variables characteristics

In this experiment, the two different arm movements served as independent variables. The arm movements were categorized as butterfly arm swing or forward arm swing. In the butterfly arm swing, the swimmer swung their arms back, up, and forward in a circular motion after the starting signal^15^. In contrast, during the forward arm swing, the arms were released from the block and swung directly forward^16^. A specific description of the anatomical characteristics of both starts is presented in Tables 2 and 3. The starting position of both types of start was characteristic of a kick-start. The subject’s weight on the block during this test was in the front-weight position. The swimmers were instructed to lean their shoulders forward in front of their hands, moving the center of mass forward toward the front of the block. The grab bars on the sides of the platform allowed swimmers to grab and push.

**Table 2:**
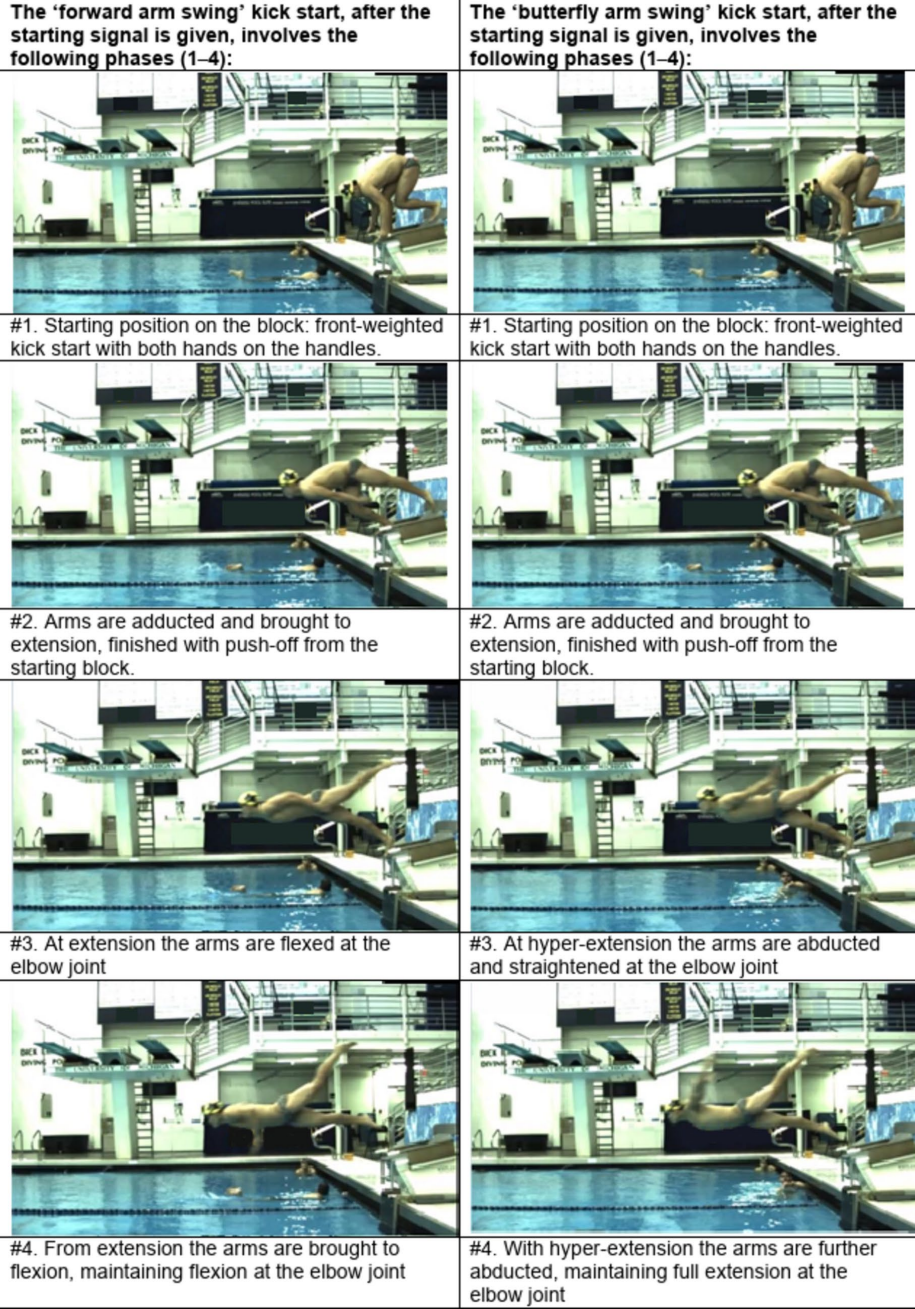
Forward arm swing and butterfly arm swing characteristics (phases 1–4).

**Table 3:**
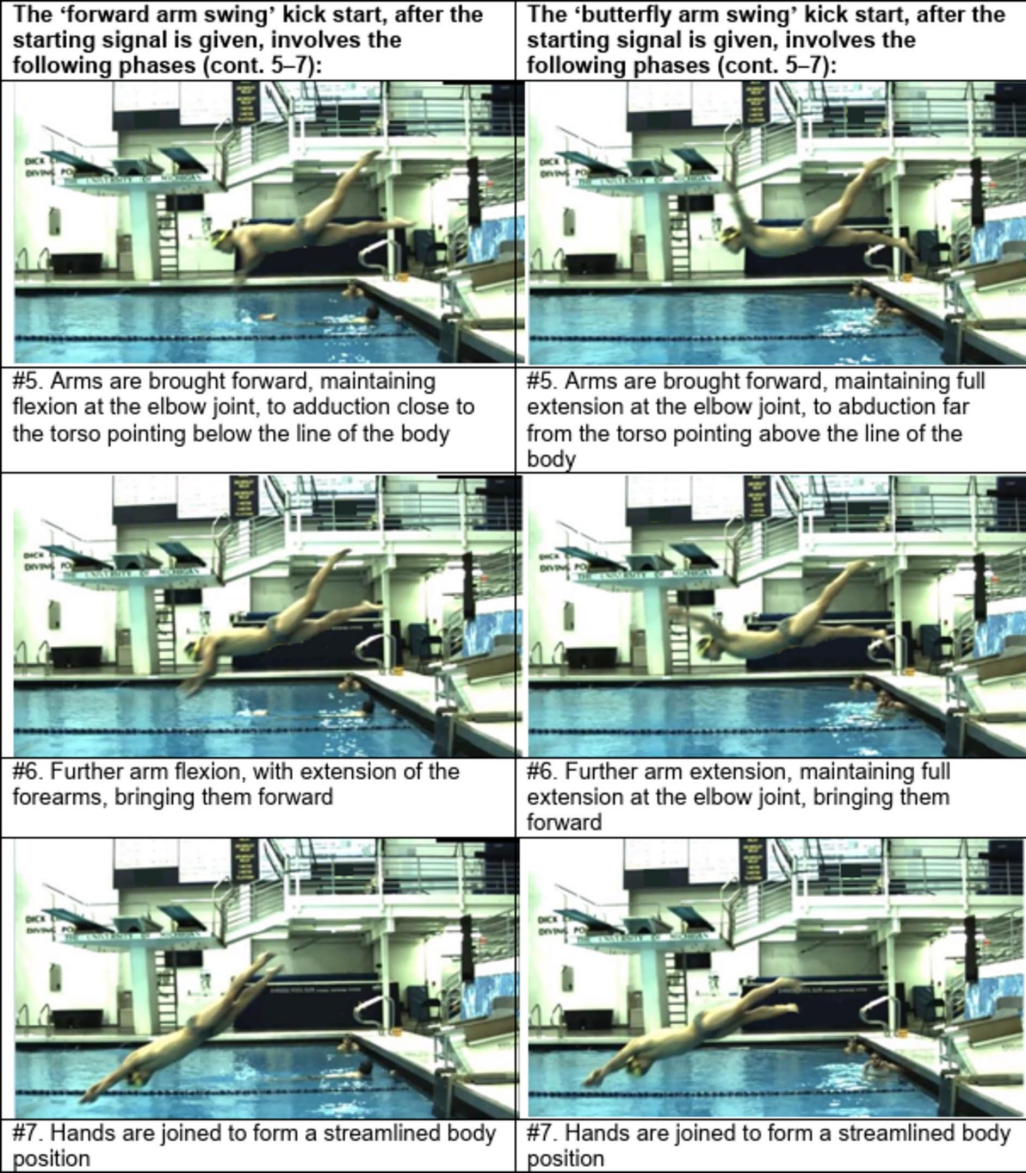
Forward arm swing and butterfly arm swing characteristics (continuation: phases 5–7).

### Dependent variables estimation

Video footage was automatically digitized using the Kistler System. The following kinematic and kinetic dependent variables have been defined according to the manual of Kistler Instrumente AG Group^29^ and categorized into the following groups: calculated from ground reaction force, above the water, and underwater, which are presented in Table 4.

**Table 4:** Terms and definitions of the dependent variables used to characterize the structure of the swimming start.

Calculated from ground reaction force		
1	**AC**_**AV**_ [m⸱s^− 1^[⸱s]^−1^]	Average horizontal acceleration was defined as the ratio of the swimmer’s horizontal center of gravity (COG) velocity at take-off to the time elapsed from the start signal until the swimmer’s feet lost contact with the starting block.
2	**W**_**TOT**_ [joules⸱kg^− 1^]	Mechanical total work was defined as the mechanical work produced by the swimmer from the start signal until take-off, normalized to body mass, and calculated as the product of the swimmer’s average mechanical power output during the block phase and the block time.
3	**P**_**AV**_ [W⸱kg^− 1^]	Average power was defined as the power produced from the starting signal to when the swimmer left the starting block, normalized to the swimmer’s body weight and calculated as the product of (absolute force ⸱ absolute velocity)⸱body mass^**−** 1^.
4	**P**_**PK**_ [W⸱kg^− 1^]	Peak power was defined as the maximum value of power measured during the block phase (from the starting signal to when the swimmer left the starting block), normalized to the swimmer’s body weight.
5	**F**_**RES.AV**_ [N⸱BW^− 1^]	Average resultant force was defined as the value of force exerted over the starting block during the block phase (sum of resultant forces on the front and rear plates, corrected for block angle) minus the resultant force on the grab bar, normalized to the swimmer’s body weight.
6	**F**_**RES.PK**_ [N⸱BW^− 1^]	Peak resultant force was defined as the maximum value of force exerted over the starting block by the swimmer during the block phase (sum of resultant forces on the front and rear plates, corrected for block angle) minus the resultant force on the grab bar, normalized to the swimmer’s body weight.
7	**F**_**H.PK**_ [N⸱BW^− 1^]	Peak horizontal force was defined as the maximum value of force exerted over the starting block in the horizontal direction by the swimmer during the block phase (sum of resultant forces on the front and rear plates, corrected for block angle) minus the resultant force on the grab bar, normalized to the swimmer’s body weight.
8	**F**_**V.PK**_ [N⸱BW^− 1^]	Peak vertical force was defined as the maximum value of force exerted over the starting block in the vertical direction by the swimmer during the block phase (sum of resultant forces on the front and rear plates, corrected for block angle) minus the resultant force on the grab bar, normalized to the swimmer’s body weight.
9	**F**_**R.RES.AV**_ [N⸱BW^− 1^]	Average resultant force on rear plate was defined as the value of force exerted over the rear plate (corrected for block angle) combined with the resultant force on the grab bar of the starting block during the block phase, normalized to the swimmer’s body weight.
10	**F**_**R.H.PK**_ [N⸱BW^− 1^]	Peak horizontal force on rear plate was defined as the maximum value of horizontal force exerted on the rear plate (corrected for the block angle) combined with the horizontal force applied to the grab bar during the block phase, normalized to the swimmer’s body weight.
11	**F**_**R.V.PK**_ [N⸱BW^− 1^]	Peak vertical force on rear plate was defined as the maximum value of vertical force exerted on the rear plate (corrected for the block angle) combined with the vertical force applied to the grab bar during the block phase, normalized to the swimmer’s body weight.
12	**F**_**F.RES.AV**_ [N⸱BW^− 1^]	Average resultant force on front plate was defined as the value of force exerted on the front plate (corrected for block angle) combined with the resultant force applied to the grab bar during the block phase, normalized to the swimmer’s body weight.
13	**F**_**F.H.PK**_ [N⸱BW^− 1^]	Peak horizontal force on front plate was defined as the maximum value of horizontal force exerted on the front plate (corrected for the block angle) combined with the horizontal force applied to the grab bar during the block phase, normalized to the swimmer’s body weight.
14	**F**_**F.V.PK**_ [N⸱BW^− 1^]	Peak vertical force on front plate was defined as the maximum value of vertical force exerted on the front plate (corrected for the block angle) combined with the vertical force applied to the grab bar during the block phase, normalized to the swimmer’s body weight.
15	**T**_**ON.BLOCK**_ [s]	Time on block was defined as the duration from the starting signal to take-off, where take-off is defined as the last sample at which the sum of vertical and horizontal forces exerted on the starting block is greater than or equal to 100 N.
**Above the water**		
16	**V**_**H.TAKE−OFF**_ [m⸱s^− 1^]	Take-off horizontal velocity was defined as the velocity obtained by integrating acceleration (a = F⸱m^**−** 1^). This represents the change in horizontal displacement as the swimmer leaves the block.
17	**V**_**V.TAKE−OFF**_ [m⸱s^− 1^]	Take-off vertical velocity was defined as the velocity obtained by integrating acceleration (a = F⸱m^**−** 1^). This represents the change in vertical displacement as the swimmer leaves the block.
18	**ANG**_**TAKE−OFF**_ [deg]	Take-off angle of COG was defined as the angle at which the swimmer’s center of mass is relative to the block during take-off. Digitized at the COG at take-off and calculated as arctangent of the vertical level at take-off divided by the horizontal level at take-off.
19	**DIS**_**H.ENT**_ [m]	Horizontal distance of water entry was defined as the distance from the start wall to the top of the head at water entry.
20	**SIZ**_**ENT.HOLE**_ [m]	Entry hole size was defined as the size of the hole created by the swimmer as they entered the water, digitized at the points where the hands contacted the water surface (far edge of the hole) and where the feet entered the water (rear edge of the hole).
21	**ANG**_**ENT**_ [deg]	Angle of water entry was defined as the arctangent of the slope of the center of gravity (COG) trajectory at water level while the swimmer’s hands were in contact with the water surface (entry point).
**Underwater**		
22	**V**_**AV.UND**_ [m⸱s^− 1^]	Average underwater velocity was defined as the average velocity of the swimmer during the underwater phase of the start, from hand entry into the water to breakout (top of the head surfacing).
23	**V**_**AV.UND.0−5**_ [m⸱s^− 1^]	Average velocity from the starting signal to 5 m was defined as the swimmer’s mean velocity over this distance.
24	**DIS**_**UND**_ [m]	Underwater horizontal distance was defined as the distance the swimmer traveled during the underwater phase of the start, from hand entry into the water to breakout (top of the head surfacing).
25	**DEP**_**H.MAX**_ [m]	Distance of maximum depth was defined as the horizontal distance from the start wall to the center of the head at its deepest point upon water entry, prior to ascent.
26	**DEP**_**V.MAX**_ [m]	Maximum depth was defined as the vertical position of the center of the head at its deepest point upon water entry, prior to ascent.
27	**T**_**5**_ [s]	Time from the starting signal to 5 m was defined as the time for the swimmer’s head to travel from the start signal to the 5 m mark, based on the digitized head point.
28	**T**_**7.5**_ [s]	Time from starting signal to 7.5 m was defined as the time for the swimmer’s head to travel from the start signal to the 7.5 m mark, based on the digitized head point.
29	**T**_**10**_ [s]	Time from starting signal to 10 m was defined as the time for the swimmer’s head to travel from the start signal to the 10 m mark, based on the digitized head point.
30	**T**_**15**_ [s]	Time from starting signal to 15 m was defined as the time for the swimmer’s head to travel from the start signal to the 15 m mark, based on the digitized head point.

### Statistical analyses

The quantitative study analysis was performed using a four-dimensional approach, alpha (α), power (1-β), sample size (n), effect size (d, r), and followed the accepted methodology^30^. The minimal sample size (*n* = 27) was calculated a priori using G*Power 3.1.9.2 (Franz Faul, Kiel, Germany), assuming a medium effect size (Cohen’s d = 0.50), a significance level of α = 0.05, and a statistical power of 1-β = 0.80, which is commonly considered adequate in sport sciences research. Post-hoc statistical power was calculated based on the observed Cohen’s d effect size for the differences between means (Student’s t-test), which was 0.32. The study included 12 participants, the significance level (α) was set at 0.05, and the estimated power (1-β) was 0.27^31^. Data are given as mean (M), standard deviation (SD), confidence intervals (CI95%), and the difference (Δ) between forward arm swing and butterfly arm swing values. Changes (increase or decrease) in parameters were also expressed as a dimensionless ratio of two quantities (%) with respect to the forward arm swing (denominator). The coefficient of variation (CV) (%) was used to determine the variability of the assessed parameters between two measurements. CV was interpreted as CV < 5% low variance; CV > 0.05 (or CV > 5%) high variance. CV was calculated using the following formula^32^(2):

2 $$\:\mathrm{C}\mathrm{V}=(\mathrm{s}\mathrm{a}\mathrm{m}\mathrm{p}\mathrm{l}\mathrm{e}\mathrm{s}\:_{\mathrm{S}\mathrm{D}}\cdot \mathrm{s}\mathrm{a}\mathrm{m}\mathrm{p}\mathrm{l}\mathrm{e}\mathrm{s}\:_\mathrm{M}^{-1})\cdot 100$$

where: samples _SD_ is the pooled standard deviation of both samples; samples _M_ is the pooled mean of both samples.

The distribution of the data set was screened for normality using the Shapiro-Wilk test. The homogeneity of variance was assessed using Levene’s test. Variables with normal distribution (*p* > 0.05) were compared using the parametric t-Student test for dependent variables. Variables with no normal distribution (*p* < 0.05) were compared using their non-parametric alternative, i.e., the Wilcoxon test for dependent variables. For each subject, the statistical tests used the average values from two repetitions for the given starting variant (two forward and two butterfly-arm swing observations). Calculations and processing of data were performed with the IBM SPSS Statistics version 26 software package (IBM, Inc., USA). Significance was set at an α level of 0.05 for all statistical procedures, with p-values provided for all results. Results and reporting style followed the recommendations of the American Medical Association Manual of Style^33^.

Effect sizes for the t-Student test were calculated using Cohen’s following formula^34^(3):

3 $$\:\mathrm{d}=(\mathrm{C}\mathrm{S}_\mathrm{M}\:-\:\mathrm{M}\mathrm{S}_\mathrm{M})\cdot \left(\mathrm{s}\mathrm{a}\mathrm{m}\mathrm{p}\mathrm{l}\mathrm{e}\mathrm{s}_{\:\mathrm{S}\mathrm{D}}\right)^{-1}$$

where: CS_M_ and MS_M_ are means of the given group of samples, and samples _SD_ are pooled standard deviations for the two groups of samples.

Effect sizes for the Wilcoxon test were calculated using Rosenthal’s formula^35^(4):

4 $$\:\mathrm{r} = \mathrm{Z}\cdot (\surd\:\mathrm{N})^{-1}$$

where: Z is the standardized value of the Wilcoxon test, and N is a number of pairs used to compute.

Effect sizes for Cohen’s d are interpreted as 0.2 (small), 0.5 (medium), and 0.8 (large)^36^. Effect sizes for Rosenthal’s r are interpreted as 0.1 (small), 0.3 (medium), and 0.5 (large)^37^.

Spearman’s (rho) nonparametric correlation analysis determined the relationships among all variables for the two types of arm swing to establish the features that are correlated with a 15 m swim time (T_15_). The correlation coefficients (*ρ*) were classified as very weak to negligible (0-0.2), weak (0.2–0.4), moderate (0.4–0.7), strong (0.7–0.9), and very strong (0.9-1.0)^38^.

To build models predicting the 15 m time for each type of arm swing, 30 variables were used. The set of variables included one dependent variable and 29 independent variables. The dependent variable was the time to 15 m (y). Independent variables included kinematic and kinetic parameters (x_0_–x_28_). The Least Absolute Shrinkage and Selection Operator (adaptive LASSO) regression model was adopted. This method can consistently select relevant variables, reduce estimation bias, improve prediction accuracy, prevent model overfitting, and perform well even with limited sample sizes^39^. Furthermore, LASSO can perform effectively without satisfying the typical assumptions of classical linear regression^40^. LASSO regression minimizes a cost function that combines the mean squared error (MSE) with an L1 penalty on the regression coefficients. The cost function is given by the formula^41^(5):

5 $$\:\mathrm{J}\left(\mathrm{w}\right)=\left(1 \cdot\right(2\cdot \mathrm{n})^{-1})\cdot \left|\right|\mathrm{y}-\mathrm{X}\mathrm{w}\left|\right|^2_2\:+ \alpha\:\cdot \left|\right|\mathrm{w}\left|\right|_1$$

where: n is the number of samples (observations), y is the vector of target values, X is the feature matrix (explanatory variables), w is the vector of regression coefficients, α is the regularization parameter, || · ||^2^_2_ denotes the squared Euclidean norm (the square of the vector’s length), || · ||_1_ denotes the L1 norm (the sum of the absolute values of the coefficients).

LASSO estimates the value of the dependent variable as a linear combination of features (independent variables)^41^(6):

6 $$\:\mathrm{y}=\mathrm{w}_0+\mathrm{w}_1\mathrm{x}_1+\mathrm{w}_2\mathrm{x}_2+\dots+\mathrm{w}_\mathrm{p}\mathrm{x}_\mathrm{p}$$

where: y is the predicted value of the dependent variable, w_0_ is the intercept (bias term), w_1_, w_2_, …, w_p_ are the regression coefficients, x_1_, x_2_, …, x_p_ are the independent variables (features).

Before performing LASSO regression, the data were scaled using the *StandardScaler* tool from the Scikit-learn library. *StandardScaler* standardizes features by transforming each variable to have a mean of 0 and a standard deviation of 1. This type of scaling is particularly important for regularization-based models, such as LASSO, as it ensures the comparability of the weights assigned to individual variables^42^. The modelling was performed with the Python 3.9.7 software package (Python Software Foundation, Wilmington, USA).

Due to the limited sample size, for the selection and evaluation of the LASSO regression models, the Leave-One-Out Cross-Validation (LOOCV) procedure was employed, which maximizes the use of available data and provides reliable and overfitting-resistant estimates^43^. In LOOCV, the dataset is split such that, in each iteration, one observation serves as the validation set while the remaining observations form the training set. This process was repeated for each observation, ensuring that every case served once as the test set.

The regularization parameter α was tuned manually by testing values in the range from 0.01 to 1.00, with a step size of 0.01. For each tested α, two error metrics were computed, i.e., RMSE_CV_ (Root Mean Square Error of Cross-Validation), representing the prediction error based on LOOCV, and RMSE_T_ (Root Mean Square Error on Training), representing the model’s fitting error on the training set. The model corresponding to the lowest RMSE_CV_ was selected as the best-performing model, following the procedure described by Przednowek et al.^44^. After selecting the optimal model, the final LASSO regression was retrained on the entire dataset to determine the final set of important variables influencing time to 15 m.

## Results

The numerous analyses conducted were based on the standard parameters of kinematics and kinetics structure of the swimming start technique. The selected parameters were an objective source of evidence used in quantitative and qualitative evaluation of swimming start performances.

### Comparison analysis

Table 5 shows a comparison of selected variables between the butterfly arm swing and the forward arm swing. Among variables calculated from ground reaction force, the butterfly arm swing was characterized by a greater mechanical total work (*p* = 0.018), average power (*p* = 0.039), peak power leaving the block (*p* = 0.001), higher average resultant force (*p* = 0.015), and peak resultant force (*p* = 0.016), greater peak horizontal force (*p* = 0.019) and peak vertical force (*p* = 0.047), higher average resultant force on rear plate (*p* = 0.010), peak horizontal force on rear plate (*p* = 0.011), and peak vertical force on rear plate (*p* = 0.031), and higher average resultant force on front plate (*p* = 0.043) (Table 5).

Among the above the water variables, the butterfly arm swing compared to the forward arm swing was characterized by a greater take-off vertical velocity (*p* = 0.003), take-off angle (*p* = 0.004), horizontal distance at water entry (*p* < 0.001), and a larger size of the entry hole (*p* < 0.014) (Table 5).

Finally, among underwater variables, the butterfly arm swing compared to the forward arm swing was characterized by greater average velocity of underwater phase (*p* = 0.048), shallower maximum depth (*p* = 0.019), and shorter time to 7.5 m (*p* = 0.028), 10 m (*p* = 0.008), and 15 m (*p* < 0.001) (Table 5).

**Table 5:** Comparison of selected variables between forward and butterfly arm swing (t-Student and Wilcoxon tests outcomes).

Dependent variables		Forward arm swing			Butterfly arm swing			Comparison analysis						
		M	SD	CI95%	M	SD	CI95%	Δ	SD Δ	% diff	CV	test value (t, Z)	*p*	ES (d, *r*)
**Calculated from ground reaction force**														
1	**AC**_**AV**_ [m⸱s^− 1^[⸱s]^−1^]	6.38	0.49	6.16–6.60	6.49	0.36	6.33–6.65	0.10	0.37	1.64	1.15	-0.97	0.354	0.23
2	**W**_**TOT**_ [joules⸱kg^− 1^]	16.38	1.64	15.67–17.09	17.10	1.55	16.45–17.75	0.73	0.91	4.44	3.07	-2.76	0.018*	0.44
3	**P**_**AV**_ [W⸱kg^− 1^]	24.09	3.40	22.64–25.54	25.38	2.44	24.34–26.41	1.28	1.90	5.33	3.67	-2.35	0.039*	0.42
4	**P**_**PK**_ [W⸱kg^− 1^]	64.88	9.83	60.64–69.11	69.38	8.29	65.84–72.91	4.50	3.59	6.94	4.74	-4.35	0.001*	0.47
5	**F**_**RES.AV**_ [N⸱BW^− 1^]	1.50	0.15	1.43–1.57	1.55	0.15	1.49–1.61	0.05	0.06	3.25	2.26	-2.87	0.015*	0.31
6	**F**_**RES.PK**_ [N⸱BW^− 1^]	2.21	0.20	2.12–2.29	2.29	0.16	2.22–2.36	0.09	0.11	3.91	2.71	-2.83	0.016*	0.46
7	**F**_**H.PK**_ [N⸱BW^− 1^]	1.34	0.16	1.27–1.41	1.39	0.14	1.33–1.45	0.05	0.07	4.02	2.78	-2.76	0.019*	0.35
8	**F**_**V.PK**_ [N⸱BW^− 1^]	1.85	0.20	1.76–1.93	1.92	0.16	1.86–1.99	0.08	0.12	4.31	2.99	-2.24	0.047*	0.42
9	**F**_**R.RES.AV**_ [N⸱BW^− 1^]	0.64	0.06	0.61–0.66	0.66	0.05	0.64–0.68	0.02	0.02	3.27	2.28	-3.11	0.010*	0.36
10	**F**_**R.H.PK**_ [N⸱BW^− 1^]	1.03	0.13	0.97–1.08	1.07	0.12	1.02–1.12	0.05	0.05	4.47	3.09	-3.03	0.011*	0.34
11	**F**_**R.V.PK**_ [N⸱BW^− 1^]	1.04	0.12	0.98–1.09	1.10	0.11	1.06–1.15	0.07	0.10	6.60	4.52	-2.47	0.031*	0.56
12	**F**_**F.RES.AV**_ [N⸱BW^− 1^]	0.91	0.16	0.84–0.98	0.95	0.17	0.88–1.02	0.04	0.06	4.39	3.04	-2.29	0.043*	0.23
13	**F**_**F.H.PK**_ [N⸱BW^− 1^]	0.759	0.09	0.72–0.80	0.755	0.07	0.72–0.79	-0.004	0.04	-0.60	0.43	0.36	0.725	0.05
14	**F**_**F.V.PK**_ [N⸱BW^− 1^]	1.31	0.17	1.23–1.38	1.34	0.18	1.26–1.41	0.03	0.07	2.33	1.63	-1.54	0.153	0.17
15	**T**_**ON.BLOCK**_ [s]	0.69	0.05	0.66–0.71	0.68	0.04	0.66–0.69	-0.01	0.02	-1.40	1.00	1.43	0.180	0.20
**Above the water**														
16	**V**_**H.TAKE−OFF**_ [m⸱s^− 1^]	4.35	0.20	4.26–4.44	4.37	0.21	4.29–4.46	0.02	0.14	0.53	0.37	-0.56	0.584	0.11
17	**V**_**V.TAKE−OFF**_ [m⸱s^− 1^]	0.04	1.18	-0.46–0.54	0.33	1.14	-0.14–0.81	0.29	0.27	759.14	111.93	-3.78	0.003*	0.24
18	**ANG**_**TAKE−OFF**_ [deg]	0.83	14.91	-5.45–7.12	4.46	14.35	-1.50–10.42	3.63	3.49	435.00	96.88	-3.60	0.004*	0.24
19	**DIS**_**H.ENT**_ [m]	3.06	0.12	3.01–3.11	3.21	0.10	3.16–3.25	0.15	0.06	4.78	3.30	-9.07	< 0.001*	1.25
20	**SIZ**_**ENT.HOLE**_ [m]	0.63	0.20	0.54–0.72	0.71	0.16	0.64–0.78	0.08	0.10	12.90	8.57	-2.91	0.014*	0.43
21	**ANG**_**ENT**_ [deg]	49.54	1.36	48.89–50.19	49.42	1.94	48.57–50.26	-0.13	1.63	-0.25	0.18	0.27	0.795	0.07
**Underwater**														
22	**V**_**AV.UND**_ [m⸱s^− 1^]	2.43	0.17	2.36–2.51	2.46	0.15	2.40–2.52	0.03	0.04	1.05	0.74	-2.23	0.048*	0.15
23	**V**_**AV.UND.0−5**_ [m⸱s^− 1^]	3.55	0.11	0.09–0.17	3.62	0.13	0.10–0.19	0.07	0.11	1.86	1.31	-2.16	0.054	0.50
24	**DIS**_**UND**_ [m]	8.54	1.67	7.83–9.25	8.31	1.45	7.70–8.92	-0.23	0.41	-2.71	1.97	-0.86	0.388	0.25^
25	**DEP**_**H.MAX**_ [m]	6.08	0.79	5.73–6.43	6.24	0.82	5.87–6.60	0.15	0.26	2.54	1.77	-2.05	0.065	0.18
26	**DEP**_**V.MAX**_ [m]	0.89	0.21	0.98 − 0.80	0.81	0.17	0.88 − 0.74	-0.08	0.10	9.03	6.69	-2.76	0.019*	0.41
27	**T**_**5**_ [s]	1.41	0.05	1.39–1.43	1.39	0.05	1.36–1.41	-0.02	0.03	-1.45	1.07	2.14	0.056	0.39
28	**T**_**7.5**_ [s]	2.32	0.11	2.27–2.36	2.26	0.11	2.22–2.31	-0.05	0.07	-2.21	1.58	2.52	0.028*	0.46
29	**T**_**10**_ [s]	3.61	0.14	3.55–3.67	3.54	0.16	3.48–3.61	-0.06	0.05	-1.71	1.22	-2.67	0.008*	0.77^
30	**T**_**15**_ [s]	6.30	0.21	6.21–6.39	6.20	0.22	6.11–6.29	-0.11	0.06	-1.69	1.20	6.62	< 0.001*	0.48

Note: M - mean, SD - standard deviation, CI95% − 95% confidence interval, Δ and % difference with respect to the forward arm swing, sd Δ - the standard deviation of the difference between two means, CV - coefficient of variation, test value (t, Z) - results of the dependent tests: parametric t-Student (t) and the nonparametric Wilcoxon (Z), *significant difference at *p* < 0.05 vs. forward and butterfly-arm swing value, ES - effect sizes for the t-Student (Cohen-d), and ^ the Wilcoxon (Rosenthal-r). Detailed variable descriptions (see Table 4).

### Correlation analysis

Spearman’s analysis indicated significant correlations with time to 15 m. Among the ground reaction force parameters for both types of arm swing, significant correlations were observed with average resultant force on rear plate, peak horizontal force on rear plate, and peak vertical force on rear plate (*p* < 0.05). For the forward arm swing, significant correlations were found with peak horizontal force on front plate and peak vertical force on front plate. For the butterfly arm swing, significant correlations were found with average acceleration on the block phase, average power, and average resultant force on front plate (*p* < 0.05).

Among the above the water parameters for the forward arm swing, significant correlations were observed with take-off vertical velocity and take-off angle (*p* < 0.05). For the butterfly arm swing, a significant correlation was observed with horizontal distance at water entry (*p* < 0.05).

Among the underwater parameters for both types of arm swings, significant correlations were found with average velocity of underwater phase, average velocity of underwater phase to 5 m, time to 7.5 m, 10 m, and 15 m (*p* < 0.05) (Table 6).

**Table 6:** Spearman correlation coefficients estimated between Time_[15]_ and kinematics and kinetics variables for the two types of arm swings.

Dependent variables		T_15_ [s]					
		Forward arm swing				Butterfly arm swing	
		ρ		*p*		ρ	*p*
**Calculated from ground reaction force**							
1	**AC**_**AV**_ [m⸱s^− 1^[⸱s]^−1^]		-0.218		0.305	-0.641	0.001*
2	**W**_**TOT**_ [joules⸱kg^− 1^]		-0.328		0.117	-0.144	0.501
3	**P**_**AV**_ [W⸱kg^− 1^]		-0.364		0.080	-0.479	0.018*
4	**P**_**PK**_ [W⸱kg^− 1^]		-0.282		0.182	-0.359	0.085
5	**F**_**RES.AV**_ [N⸱BW^− 1^]		-0.052		0.810	0.102	0.634
6	**F**_**RES.PK**_ [N⸱BW^− 1^]		-0.265		0.210	-0.371	0.074
7	**F**_**H.PK**_ [N⸱BW^− 1^]		-0.380		0.067	-0.120	0.577
8	**F**_**V.PK**_ [N⸱BW^− 1^]		-0.007		0.976	0.057	0.790
9	**F**_**R.RES.AV**_ [N⸱BW^− 1^]		-0.662		< 0.001*	-0.727	< 0.001*
10	**F**_**R.H.PK**_ [N⸱BW^− 1^]		-0.479		0.018*	-0.528	0.008*
11	**F**_**R.V.PK**_ [N⸱BW^− 1^]		-0.685		< 0.001*	-0.621	0.001*
12	**F**_**F.RES.AV**_ [N⸱BW^− 1^]		0.363		0.081	0.444	0.030*
13	**F**_**F.H.PK**_ [N⸱BW^− 1^]		0.415		0.044*	0.177	0.408
14	**F**_**F.V.PK**_ [N⸱BW^− 1^]		0.516		0.010*	0.309	0.142
15	**T**_**ON.BLOCK**_ [s]		0.275		0.193	0.401	0.052
**Above the water**							
16	**V**_**H.TAKE−OFF**_ [m⸱s^− 1^]		0.030		0.889	-0.142	0.507
17	**V**_**V.TAKE−OFF**_ [m⸱s^− 1^]		-0.426		0.038*	-0.393	0.057
18	**ANG**_**TAKE−OFF**_ [deg]		-0.435		0.034*	-0.385	0.063
19	**DIS**_**H.ENT**_ [m]		-0.378		0.069	-0.568	0.004*
20	**SIZ**_**ENT.HOLE**_ [m]		-0.047		0.829	-0.047	0.827
21	**ANG**_**ENT**_ [deg]		-0.032		0.884	0.287	0.174
**Underwater**							
22	**V**_**AV.UND**_ [m⸱s^− 1^]		-0.560		0.004*	-0.509	0.011*
23	**V**_**AV.UND.0−5**_ [m⸱s^− 1^]		-0.641		0.001*	-0.632	0.001*
24	**DIS**_**UND**_ [m]		0.155		0.470	0.105	0.626
25	**DEP**_**H.MAX**_ [m]		0.122		0.571	-0.068	0.754
26	**DEP**_**V.MAX**_ [m]		-0.289		0.171	-0.197	0.356
27	**T**_**5**_ [s]		0.641		< 0.001*	0.750	< 0.001*
28	**T**_**7.5**_ [s]		0.779		< 0.001*	0.709	< 0.001*
29	**T**_**10**_ [s]		0.846		< 0.001*	0.838	< 0.001*

Note: *significant difference of correlation at *p* < 0.05, *ρ* - Spearman’s correlation coefficients. Detailed variable descriptions (see Table 4).

### Regression analysis

**Fig. 3:**
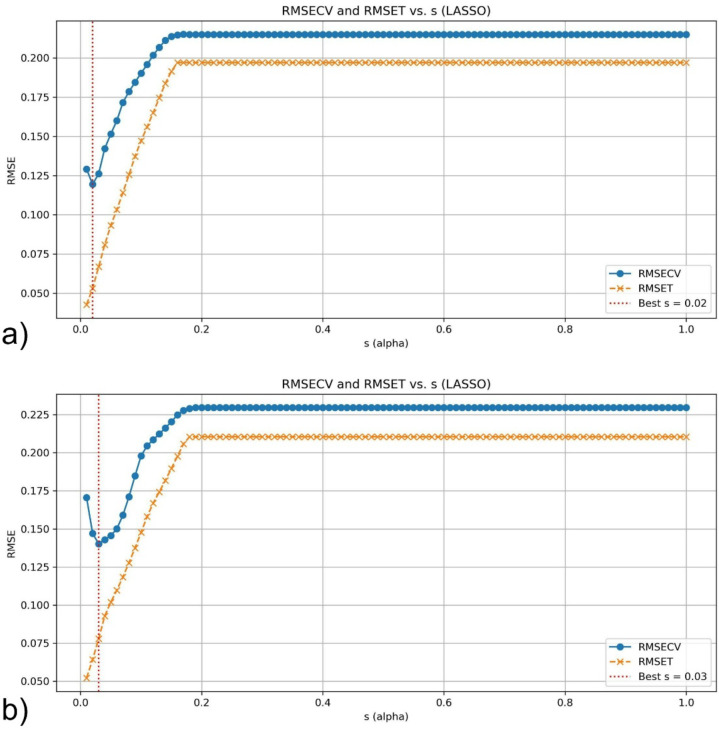
Prediction and training errors for LASSO regression; dashed line marks the best model: **a**) forward arm swing, and **b**) butterfly arm swing.

The predictor variables (independent) are presented in Eqs. (7) and (8). The LASSO method rejected the remaining variables. The model for forward arm swing was cross validated for s equal from 0.01 to 1 with s = 0.02 steps of 0.01. The best LASSO model was obtained with the error RMSE_CV_=0.119, and RMSE_T_=0.053 (Fig. 3a). Additionally, the LASSO method eliminated several predictors (coefficients equal 0), so the model has the form (only variables with coefficients ≠ 0): (7):

7 $$\:\mathrm{y}\left(\mathrm{T}_{15}\right)\:=\:-0.020\:\cdot\:\:\mathrm{F}_{\mathrm{R}\mathrm{E}\mathrm{S}.\mathrm{P}\mathrm{K}}\:+\:-0.030\:\cdot \:\:\mathrm{F}_{\mathrm{R}.\mathrm{H}.\mathrm{P}\mathrm{K}}\:+\:-0.046\:\cdot \:\:\mathrm{F}_{\mathrm{R}.\mathrm{V}.\mathrm{P}\mathrm{K}}\:+\:-0.084\:\cdot \:\:\mathrm{V}_{{\mathrm{A}\mathrm{V}.\mathrm{U}\mathrm{N}\mathrm{D}}}+ 0.062\:\cdot\:\:\mathrm{T}_{10}$$

The model for butterfly arm swing was cross validated for s equal from 0.01 to 1 with s = 0.03 steps of 0.01. The best LASSO model was obtained with the error RMSE_CV_=0.140, and RMSE_T_=0.078 (Fig. 3b). Additionally, the LASSO method eliminated several predictors (coefficients equal 0), so the model has the form (only variables with coefficients ≠ 0): (8):

8 $${\rm y\left(T_{15}\right)=\:-0.022\:\cdot\:\:AC_{AV}\:+\:0.002\:\cdot\:\:T_{ON.BLOCK}\:+\:-0.017\:\cdot \:\:SIZ_{ENT.HOLE}\:+\:-0.062\:\cdot \:\:V_{AV.UND}+0.113\:\cdot\:\:T_{10}}$$

The models for both types of arm swing show a low RMSE_T_, indicating a good fit for the data. According to Hastie et al.^45^, cross-validation provides an unbiased estimate of prediction error and is widely used to assess model generalization. Because training error is typically optimistic, comparing it with cross-validation error provides a practical check for overfitting. In this study, RMSE_CV_ and RMSE_T_ showed moderate agreement (0.119 vs. 0.053 and 0.140 vs. 0.078 for forward and butterfly-arm swing, respectively), indicating no substantial overfitting and suggesting moderate model generalization. The optimal values of the regularization parameter α were low (0.02 and 0.03) for the forward and butterfly-arm swing, respectively, implying that the model retained most variables while penalizing only the least important ones.

## Discussion

The biomechanical mechanisms using two different arm swing techniques in the swim start in elite swimmers were investigated in this study. The study demonstrated improvement in overall start performance expressed by front crawl swim time to 15 m (*p* < 0.001) when the swimmers performed the butterfly arm swing vs. the forward arm swing, which is the main finding of this research. However, this is not necessarily related to the early phase of the start, as no significant differences were found between the analyzed swim starts at the 5 m time point (*p* = 0.056). It is well known that the performance of a swim start is a combination of reaction time, vertical and horizontal force off the block, a low drag force during the underwater phase, and underwater undulatory swimming^46^. In this study, significant differences were found in most variables between the butterfly arm swing and the forward arm swing.

### Ground reaction force variables

The swimmers in the current study displayed a significant (*p* = 0.018) greater overall mechanical total work when using the butterfly arm swing. This significant difference was related to the increased average and peak power from the starting signal to take-off (*p* < 0.05). This might reflect the fact that the butterfly arm swing produced a significant increase in several horizontal and vertical force metrics during the push-off phase (*p* < 0.05). Based on research of dry-land jumps, one could speculate that the arm swing may enhance the transfer of body momentum from the upper to the lower limbs, enabling the legs to exert greater force on the starting block^47^. Previous kinetic research examining force production on the starting block has shown that higher horizontal and vertical peak forces are associated with better block performance^48^. Similarly, the ability to produce large forces on the starting block is considered a key determinant of block performance^49^. Moreover, a correlation was observed between time to 15 m and several force variables. Additionally, average acceleration on the block phase (ρ=-0.641) and average power (ρ=-0.479) significantly correlated with time to 15 m. These results support the findings of previous research by Lyttle et al.^50^, who reported that greater leg power leads to improved start performance.

### Above the water variables

It is recognized that obtaining the highest explosive power from the block requires a compromise between the optimal movement time and the time taken to push off from the block^9^. In turn, Cheng et al.^51^ reported that ground contact duration and total work performed are greater with arm swing. This suggests that performing the butterfly arm swing might have resulted in a longer time on the block. Interestingly, in this study, the time from the starting signal to take-off in both arm swings was not significantly different (*p* = 0.180). It is possible that, despite using two different types of arm swing, the swimmers’ experience allowed them to optimize their time on the block in order to maximize take-off velocity. The higher ability to generate take-off power and the strength accumulated as a result of the butterfly arm swing in the muscles were utilized in the form of high take-off velocity. The results show that swimmers generated significantly greater take-off vertical velocity (*p* = 0.003) when using the butterfly arm swing. Perhaps in this case, the hands, which play a role in pulling the body toward the starting block^52^, did not suppress the upward force generated by the feet during take-off.

It is considered that greater vertical velocity can extend flight and benefit overall start performance^53^. However, an excessively high vertical component of the take-off limits the maximization of the horizontal component, negatively affecting horizontal velocity during the flight phase, highlighting the need to balance the trade-off between vertical and horizontal components^54^. In the current study, swimmers, when using the butterfly arm swing, achieved significantly greater flight distance (*p* < 0.001), which was also significantly correlated with time to 15 m (ρ=-0.568). As air resistance is less than water resistance, it is advantageous to maximize the distance where the swimmer breaks the water surface^6^. The current results are also in line with a study by Breed and McElroy^55^, which showed that increasing flight distance was a key component of start performance. Similarly, Ruschel et al. (2007) reported that flight distance determines starting performance (*r*=-0.482). It should be emphasized that, in the butterfly arm swing, a farther flight distance also equated to a significantly bigger size of the hole created by the swimmer as they entered the water (*p* = 0.014). It was most likely related to a significantly larger (*p* = 0.004) angle during the take-off phase in the butterfly arm swing (4.46 deg.) compared to the forward arm swing (0.83 deg.). Previous studies showed that the take-off angle is very important in start performance. Takeda et al.^56^ determined the take-off angle by body angle at the take-off. These authors showed a significant positive correlation (*r* = 0.71, *p* < 0.05) between the take-off angle and the mechanical work. As the angle increased, the mechanical work also increased. The authors concluded that swimmers should select a lower take-off angle of around 0 degrees. In the current study, the mechanical total work was significantly higher (*p* = 0.018) during the butterfly swing, which corresponds to the cited authors.

### Underwater variables

The underwater phase is the longest phase of the start^57^, with the highest velocity compared with free swimming^58^ and contributes 84% to the total time taken to reach 15 m^59^. Consequently, it is worth noting that despite the larger water entry area (*p* = 0.014), the swimmers who used butterfly arm swing did not lose speed due to impact with the water, and they generated a significantly greater average underwater velocity (*p* = 0.048). This result suggests that achieving high speed during the flight phase could compensate for hydrodynamic drag, which increases proportionally to increasing body surface area during water entry^60^. A difference between the two types of starts in the deepest point of the swimmer upon entry into the water before ascent has also been observed. The forward arm swing created an immersion depth of 0.89 m, while the butterfly arm swing was shallower with an average of 0.81 m (*p* = 0.019). It is recognized that swimmers should perform their glides at approximately 0.4–0.6 m underwater to gain maximum drag reduction and maximization of underwater velocity benefits^61^. Similarly, the optimal glide depth was considered to range from 0.25 m to 0.75 m^62^. Pereira et al.^63^ investigated the underwater phase and showed significant correlations between the maximum depth reached during the glide (*r* = 0.515) and the average velocity of the underwater phase (*r*=-0.645) to 15 m. The butterfly arm swing resulted in a shallower depth closer to the recommended underwater range, which may enhance the benefits of the underwater phase. Furthermore, it has been proven that the way the push-off is performed affects the swimmer’s behavior during the flight and angle at the moment of water contact^64^. Although the take-off angle and take-off vertical velocity were significantly different between the forward arm swing and butterfly arm techniques (*p* < 0.05), in the current study, there was no difference between the two types of arm technique (*p* = 0.795) in water entry angle. This suggests that variations in push-off mechanics and flight parameters did not translate into changes in the angle at which the body enters the water.

### Temporal variables

The time to 15 m has been reported to be an ideal location to set the finish of the start phase distance and a critical component of overall swimming performance^65^. Additionally, time to 10 m was a strong predictor of start performance^66^. In this study, a 15 m distance was consciously used to assess start performance. The current study showed that the swimmers remained significantly faster over 15 m when using the butterfly swing (*p* < 0.001). Furthermore, the swimmers who used the butterfly arm swing recorded significantly faster from start signal to 7.5 m and 10 m (*p* = 0.028, *p* = 0.008, respectively) versus the forward arm swing. However, other authors report that about 83% of the variance in time to 5 m results from block time and flight distance and recommend using a shorter distance to better identify the on-block phase^6^. In this study, no significant differences were found on the 5 m time (*p* = 0.056) coupled with small effect sizes (0.39), which was confirmed by the non-significant (*p* = 0.054) average velocity from the start to 5 m, accompanied by medium effect sizes (0.50), which may indicate a trend toward significance. It means that when the swimmer reached 5 m, the advantage of the take-off phase of the butterfly arm swing over the forward arm swing technique decreased. However, if a swimmer covers a given distance in the same amount of time using two different start techniques but reaches that point with a higher instantaneous velocity in one of them, the higher velocity start is clearly the better start^9^. Given that races can be decided by as little as 0.01 s, the additional velocity achieved with an on block phase may provide a meaningful advantage. Although in this study, instantaneous velocity was not evaluated and tested statistically. One possible explanation can be derived from a hydrodynamic perspective. A swimmer achieving a higher take-off velocity will experience greater drag, which increases approximately proportional to the cube of the velocity and not the square^67^, upon entering the water. Consequently, any advantage gained during the block phase may dissipate by 5 m. This suggests that factors unrelated to arm swing technique, such as underwater propulsion, could have also contributed to the observed outcomes in this study. That is not to say the current data is not useful. All biomechanical changes over 5 m could be closely related and affect the trajectory of the swimmer during the underwater phase, which in turn has implications for the amount of drag acting on the swimmer^68^ and, ultimately, the time to 15 m. Moreover, although these are only assumptions, borderline significance values of time and velocity to 5 m may indicate an increase in significance with a larger sample size.

Regarding the relationship between the variables, it was confirmed that the average velocity to 5 m (*ρ*=-0.632), average velocity of the entire underwater phase (*ρ*=-0.509), and time to 5 m (*ρ* = 0.750), 7.5 m (*ρ* = 0.709), and 10 m (*ρ* = 0.838) directly influenced the time to 15 m. These outcomes corresponded with Ruschel et al.^69^, who showed that a 15 m start time was significantly correlated with average velocity of the underwater swimming phase (*r*=-0.645). The remaining temporal correlations appear logical, as faster times over 7.5 m, and 10 m would be expected to result in a faster time to 15 m.

### Regression analysis

The mechanisms behind the link between arm swing and starting performance (e.g., time to 15 m) are multifaceted. According to the LASSO regression analysis, it was verified that the combination of some ground reaction force metrics such as average acceleration on the block phase, time on block, and above the water parameter e.g. size of the entry hole, with underwater variables like average velocity of underwater phase and time to 10 m, were the ones that had a greater effect on the total time in 15 m in butterfly arm swing. In turn, for forward arm swing, GRF metrics such as peak resultant force, peak horizontal force on rear plate, and peak vertical force on rear plate, with some underwater parameters, e.g. average velocity of underwater phase, and time to 10 m, determined the time to 15 m. These findings are consistent with previous studies that applied regression analysis and identified key predictors of 15 m time among above the water start parameters, including take-off horizontal velocity and time on the block, whereas during the underwater phase, the time to 10 m was identified as a key determinant^3,70^. In addition, in this study, the best equations to predict time to 15 m may be used by coaches to estimate and monitor the performance of the two analyzed start techniques in training and competition by assessing the parameters indicated in the equations. Although this regression analysis was exploratory and comparative in nature, the results may nonetheless stimulate further research on both types of arm swing. Nevertheless, the study offers an objective assessment of the area that warrants further focus. Most importantly, for swimmers and coaches, specific parameters of the start components can be improved through training. It is therefore recommended that coaches use these key parameters identified as main indicators to improve starts with the butterfly or forward arm swing performance during training sessions.

### Practical applications

From a practical standpoint, the swimmers performing a butterfly arm swing achieved a significantly faster start time over 15 m (*p* < 0.001) versus the forward arm swing. However, as discussed, the improvement in swim time to 15 m is not necessarily related to the arm swing during the early phase of the start. Even though the butterfly arm technique did not have a clear statistically significant advantage at time to 5 m over the forward arm technique, there was a trend towards higher average velocity from the start to 5 m with the butterfly arm movements (*p* = 0.054, medium effect sizes 0.50). Coaches and swimmers may consider emphasizing upper-body modifications that significantly improve power and most force-related parameters (*p* < 0.05). It can be assumed that after a familiarization period with this type of start, athletes may achieve greater improvements in the swim start if butterfly arm movement tasks are included in training programs, especially in the sprint groups in competitive swimming, where the contribution of the start is significant. Furthermore, in contrast to the forward arm swing, the advantage of the butterfly arm swing is the absence of a sudden change in the direction of the arm movement, which would require a change in the momentum vector^71^. This pattern with continuity of motion helps maintain momentum in the desired direction and may allow for more effective coordination between the upper and lower body during take-off^12^. As a result, the swimmer can produce a greater peak force exerted over the starting block during the block phase, leading to higher acceleration and take-off velocity. This was the original purpose of our study, which was designed with a combination of comprehensive statistical analyses, including difference tests, correlation analyses, and identification of determinants of start performance, with a detailed biomechanical assessment of arm swing movement during the swim start. Studies of this nature enhance the coach’s knowledge of the different methods of starts involved in key areas of the race and may aid in training program design. The results obtained can also serve as a reference for those who want to study these issues in different conditions.

### Limitations

Several potential limitations must be considered when designing future studies. Firstly, the study included a limited number of participants, which did not reach the computed minimal sample size, resulting in low statistical power and restricting the ability to draw strong conclusions. Thus, validation of the results on larger cohorts is required. However, this group consisted of experienced individuals, and statistical tests confirmed the analyses’ reliability. Secondly, the stroke used during swimming was the front crawl, and the strategies of starting in other swimming events (e.g., breaststroke) might be different from those used in this study. Thirdly, considering the swimming start, there is evidence that male and female swimmers may adopt different movement patterns to accomplish similar tasks^72^. Hence, future studies should also include female swimmers to determine how they respond to the start techniques used by male swimmers in this study. Lastly, although the butterfly arm swing achieved a significantly faster start time over 15 m, this improvement was not necessarily related to the arm movement itself, because the first 5 m primarily reflect the swimmer’s performance on the starting block^6^. Further studies could be conducted to overcome the limitations listed above, and a deeper understanding of the role of arm movements in the swim start.

## Conclusions

The main finding in comparing the butterfly arm swing and the forward arm swing was a significant improvement in start time over the distance from 7.5 m to 15 m when using the butterfly arm swing. Nevertheless, there were no differences in time to 5 m, which is considered a more suitable criterion measure for assessing the early phase of the swimming start. From this perspective, improvements in 15 m time may not necessarily be attributable to the arm swing technique.

The butterfly arm swing enhanced power and several force-related parameters, leading to an increase in vertical take-off velocity. This resulted in a farther flight, albeit with a larger surface area during water entry and a deeper dive. However, the swimmers achieved a higher average underwater velocity. Any advantage due to the improvement of kinetic variables by the butterfly arm swing technique was negated by 5 m.

A significant correlation was revealed between the butterfly arm swing and the time to 15 m with several parameters calculated from ground reaction forces and underwater measurements. Similarly, in the regression analysis, several predictors of time to 15 m have been shown, including the average acceleration on the block phase, take-off time, size of the entry hole, the average underwater velocity, and time to 10 m. These parameters were strongly associated with the performance of the start with the butterfly arm swing.

Although these results do not make the butterfly arm technique the clear choice for swimmers, coaches may consider implementing the butterfly arm swing into training programs.

## Data Availability

Data cannot be shared publicly because of concerns over the risk of inadvertent disclosure of personal health information and performance information of elite athletes. Data are available from the Wroclaw University of Health and Sport Sciences Research Ethics Committee (contact via e-mail: katedra.oiz@awf.wroc.pl) or the first author (contact via e-mail: stefan.szczepan@awf.wroc.pl) for researchers who meet the criteria for access to confidential data. To proceed with permission to use the data set, it is necessary to conduct a joint investigation with the research staff.
